# Taming Tumor Glycolysis and Potential Implications for Immunotherapy

**DOI:** 10.3389/fonc.2017.00036

**Published:** 2017-03-13

**Authors:** Shanmugasundaram Ganapathy-Kanniappan

**Affiliations:** ^1^Division of Interventional Radiology, Russell H. Morgan, Department of Radiology and Radiological Sciences, Johns Hopkins University School of Medicine, Baltimore, MD, USA

**Keywords:** cancer metabolism, tumor glycolysis, immunotherapy, tumor microenvironment

## Abstract

Immune evasion and deregulation of energy metabolism play a pivotal role in cancer progression. Besides the coincidence in their historical documentation and concurrent recognition as hallmarks of cancer, both immune evasion and metabolic deregulation may be functionally linked as well. For example, the metabolic phenotype, particularly tumor glycolysis (aerobic glycolysis), impacts the tumor microenvironment (TME), which in turn acts as a major barrier for successful targeting of cancer by antitumor immune cells and other therapeutics. Similarly, in the light of recent research, it has been known that some of the immune sensitive antigens that are downregulated in cancer may also be restored or induced by cellular/metabolic stress. For instance, cancer cells downregulate the cell surface ligands such as MHC class I chain-related (MIC) protein-(A/B) that are normally upregulated in disease/pathological conditions. Noteworthy, the MHC class I chain-related protein A and B (MIC-A/B) are recognized by natural killer (NK) cells for immune elimination. Interestingly, MIC-A/B is stress inducible as demonstrated by oxidative stress and other cellular-stress factors. Consequently, stimulation of metabolic stress has also been shown to sensitize cancer cells to NK cell-mediated cytotoxicity. Taken together, data from recent reports imply that dysregulation of tumor glycolysis could facilitate induction of immune sensitive surface ligands leading to increased efficacy of antitumor immunotherapeutics. Nonetheless, dysregulated tumor glycolysis may also impact the TME and alter it from acidic, low pH into a therapeutically desirable TME that can enhance the effective infiltration of antitumor immune cells. In this mini-review, targeting tumor glycolysis has been discussed to evaluate its potential implications to enhance and/or facilitate anticancer immunity.

## Introduction

Among different cancer treatment modalities immunotherapy enjoys the advantage of antigen-dependent specific targeting of cancer cells. Although the therapeutic potential of host immune system in affecting cancer progression has long been known (1), only in the recent decades research on the development of effective immunotherapy has gained momentum. Approval of immunotherapeutics by the Food and Drug Administration (USA) further signified immunotherapy as one of the potent and viable approaches for cancer treatment (2). During the recent expansion of the list of hallmarks of cancer, Hanahan and Weinberg (3) included “immune-evasion” and the “deregulation of energy metabolism” (i.e., metabolic reprogramming also referred as “altered energy metabolism”) as additional molecular signatures of cancer. Both the “deregulated energy metabolism” and the immune evasion occupy similar chronological history in terms of their initial documentation (more than several decades ago) (4, 5), followed by decades of paucity and the recent recognition as cancer hallmarks (3). Emerging reports suggest that besides the historical coincidence, these two phenotypes may be functionally linked as well (6). This mini-review aims at understanding the role of tumor glycolysis in the context of immune evasion and to discuss potential immunotherapeutic implications of taming tumor glycolysis.

## Cancer Immune Evasion

**Figure 1:**
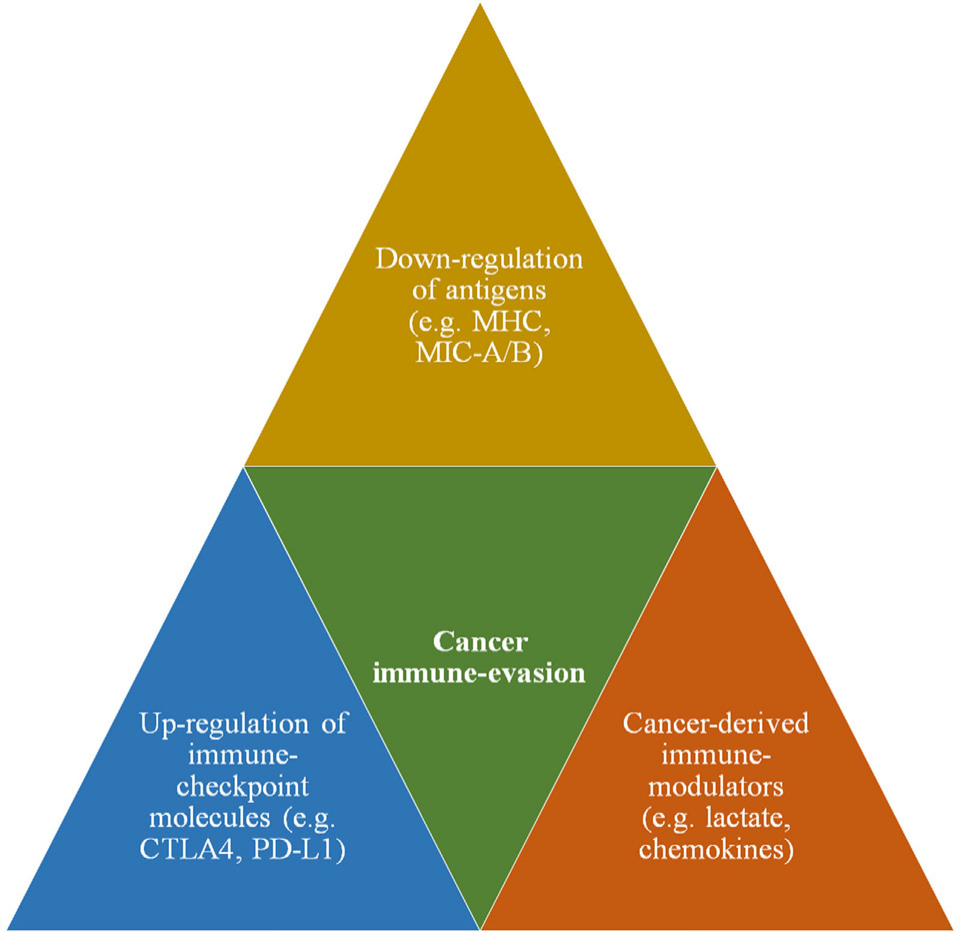
**A schematic showing major immune-evasive mechanisms that enable cancer cells to escape immune surveillance and antitumor immunity**.

Cancer cell’s propensity to escape immune surveillance is known as cancer immune evasion (3). Substantial body of evidence unequivocally demonstrate that cancer cells employ several lines of biochemical and functional alterations to evade immune detection (7). Such immune-evasive mechanisms include cancer-derived immune modulators, upregulation of immune checkpoint molecules, and downregulation of tumor-specific antigens (Figure 1). Cancer-associated immune modulation is achieved *via* certain secretory products that include but are not limited to (i) cytokines (e.g., interleukins), (ii) chemokines (e.g., SDF-1) that promote the activation of tumor-associated macrophages, and (iii) establishment of an acidic tumor microenvironment (TME) that renders majority of antitumor immune cells less efficient or non-functional (8). Next, immune checkpoint ligands prevent or suppress the antitumor activity of immune cells by inhibitory interaction with corresponding receptors on immune cells. For example, the expression of CD80 ligand on cancer cell enables it to inhibit the immune reaction of cytotoxic T lymphocytes (CTL) by binding with the specific receptor CTLA4. Similarly, the programmed death-ligand (PD-L 1,2) interferes with the antitumor function of CTLs by binding with PD-1 receptor. Finally, the downregulation of cancer-specific antigens such as major histocompatibility complex (MHC) molecules has been implicated as one of the prominent mechanisms to escape immune detection by T lymphocytes. Recent data indicate such downregulation also includes the antigens specific for natural killer (NK) cells. Experimental evidences on the ligands, MHC class I chain-related protein A or B (MIC-A/B) demonstrates that cancer cells downregulate these NKG2D ligands to prevent immune recognition by corresponding receptors on NK cells (9). Thus, antigens or ligands specific for T cells as well as NK cells are downregulated as parts of immune-escape mechanisms.

Among the innate (e.g., NK cells) and adaptive immune systems (e.g., T-cells), the latter has been under extensive preclinical and clinical investigation. Therefore, significant progress has been made in understanding the mechanistic details of T-cell-mediated antitumor immunity, leading to the development of potential therapies by harnessing T-cell’s ability to target cancer (10). For instance, the development of monoclonal antibodies (mAbs) against specific cancer antigens or tumor antigens has been very effective in specific targeting to enhance T-cell-mediated immunotherapy (11). However, such mAbs were frequently challenged with undesirable effects like the immunogenicity in patients and reduced efficiency in the recruitment of effectors cells (12). Hence, additional approaches were undertaken to overcome at least some of the impediments faced by such mAbs. Consequently, humanized chimeric antigen receptor (CAR)-T cells were developed which markedly reduced the undesirable immune reactions. However, the clinical outcomes were still less successful necessitating further research (12). Nevertheless, T-cell-dependent or -related potential therapeutics are advancing at an exponential rate toward the development of a viable strategy to achieve successful cancer treatment. Meanwhile, studies on the innate immune system such as NK cells have also been progressing remarkably to exploit potential opportunities for cancer therapy (13–15). Especially the adoptive cells transfer therapy has shown promising results and encouragement. Yet, irrespective of the type of immune therapeutics, the clinical benefits of immunotherapy have been realized primarily in hematological cancers (16, 17) and less efficient against solid malignancies.

## Tumor Glycolysis

Several elegant reviews have discussed the biology and significance of tumor glycolysis (18, 19). Hence, considering the focus of this review, the tumor glycolysis will be discussed in the context of its role and relevance in immune evasion and immunotherapy, respectively. Clinical diagnosis of cancer using positron emission tomography relies on the accelerated rate of glucose metabolism, one of the metabolic signatures of cancer cells (20). This increased glucose utilization is accomplished by a metabolic switch to glycolysis, i.e., the process of conversion of glucose into pyruvate followed by lactate production in the absence of oxidative phosphorylation (OxPhos). The pioneering work of the German scientist, Warburg (5, 21) documented for the first time that cancer cells exhibit glycolysis even in the presence of oxygen, hence popularly known as “aerobic glycolysis” or “Warburg effect.” Aerobic glycolysis or the tumor glycolysis produces fewer energy molecules (e.g., ATP) compared to the mitochondrial, OxPhos. Several elegant reviews (18, 22) have provided insights on the biological effects and advantages of such a “metabolic switch.” In fact, the metabolic switch or the “altered energy metabolism” is so frequent and common in majority, if not, all types of cancers, it has been included as one of the hallmarks of cancer (3). In this context, it is noteworthy that recent research demonstrates that aggressive phenotype of cancer is also associated with increased OxPhos, which relies on mitochondrial respiration (23, 24). However, considering the aim of this mini-review and the space limitation, the discussion on tumor metabolism will be limited to tumor glycolysis.

Clinically, tumor glycolysis has been found to be associated with some of the therapeutic challenges that impede successful cancer treatment. For example, tumor glycolysis has been implicated in therapeutic resistance (25) in chemotherapy (26), radiation therapy (27), etc. In this context, the TME has been implicated as one of the major barriers for successful targeting of cancer (28, 29). The composition of TME is primarily influenced by secretory/excretory products of cancer cells in addition to the tumor-associated fibroblasts. Lactate produced by glucose metabolism, particularly the glycolysis, is secreted/exported and remains in the TME. Hence, tumor glycolysis is one of the chief metabolic principles that orchestrate the constituents of TME. The extracellular accumulation of lactate contributes to the chemical gradient and pH of the TME (30). Experimental evidences demonstrate that the low pH or the acidity of TME either impedes the penetrability of therapeutics or renders them inactive and non-functional (30). Accordingly, effective elimination of cancer necessitates the integration of a strategy to overcome the TME barrier.

Tumor glycolysis contributes to the acidic microenvironment through the release of lactate and other low-pH ions into the extracellular milieu that in turn prevents or quenches the infiltration or efficacy of therapeutics. Thus, it is evident that tumor glycolysis invariably facilitates a protective barrier and maintains the efficient management cellular bioenergetics and redox balance in cancer. Thus, disruption of tumor glycolysis is imperative to destabilize cancer cells’ redox balance rendering them susceptible to therapeutic intervention. One of the well-investigated targets for the inhibition of tumor glycolysis is the enzyme, lactate dehydrogenase (LDH), the enzyme that catalyzes the conversion of pyruvate into lactate (31). Besides the inhibition of LDH, tumor glycolysis may also be disrupted by targeting any intermediate steps of glucose metabolism. For instance, inhibition of the enzyme hexokinase (32) or any other enzymes (33, 34) that catalyze subsequent reactions of glucose metabolism has been known to promote anticancer effects. Noteworthy, there is a distinctive advantage in targeting glycolytic steps preceding the step of lactate production. In other words, deregulation of tumor glycolysis by targeting glycolytic enzymes other than LDH may have additional desirable outcome. This is primarily due to the characteristic, “feed-back” inhibitory mechanism of glucose metabolism. Precisely, the accumulation metabolites of intermediate steps of glycolysis due to the inhibition of a particular enzyme eventually blocks or alleviates the rate of glucose catabolism in a negative feedback fashion. Thus, disruption of tumor glycolysis plausibly reduces the rate of glucose oxidation and utilization. Consequently, the energy demands of cancer cells will necessitate the utilization of alternative energy producing pathways, which is plausible due to the metabolic plasticity of cancer cells. One of the alternative pathways frequently witnessed in cancer cells to meet their energy demand is the glutamine metabolism, which relies on mitochondrial respiration. Thus, dysregulation of tumor glycolysis will necessitate cancer cells to depend on mitochondrial metabolism rendering them susceptible to any anti-mitochondrial approach using mitotropic agents (35, 36). Moreover, such unidirectional metabolic switch to mitochondrial metabolism also blocks the capacity of cancer cells to reprogram to glycolysis as the glucose consumption remains impaired due to the inhibition of glycolysis.

## Dysregulation of Tumor Glycolysis and Immunosensitivity

As discussed above, in the absence of OxPhos, lactate is the metabolic end product of tumor glycolysis. The lactate thus produced is then exported to the external milieu *via* specific transporters called monocarboxylate transporters (MCTs). Lactate is a major source of the H+ ions that contributes to the acidification of TME, although other sources of H+ ions are prevalent (37). Disruption or dysregulation of glycolysis by the inhibition of LDH has been shown to rewire the metabolism toward mitochondrial-dependent OxPhos (38). Though such a metabolic plasticity allows cancer cells to survive, the intrinsic characteristics of OxPhos have been known as undesirable for the perpetuation of tumor growth. One of the metabolic outcomes of OxPhos is the generation of free radicals collectively known as reactive oxygen species (ROS). ROS is required for the stabilization of one of the critical factors, the hypoxia inducible factor (HIF)-1 (39). Conversely, excessive accumulation of ROS is deleterious to subcellular structures and organelles (40). In fact, some of antineoplastic alkylating agents exert anticancer effects by the induction of ROS to cytotoxic levels (41). Next, as chronic accumulation of ROS is deleterious to subcellular organelles/membrane structures, it necessitates their neutralization or quenching by antioxidants (e.g., glutathione). In cancer cells, the level of antioxidants has been mitigated as the metabolic phenotype is primarily utilized for the synthesis of macromolecules that are critical for proliferation and growth. Noteworthy, antioxidants have also been implicated as potential anticancer agents as they eliminate ROS, which is required for the stabilization of HIF-1 (42). Thus, the maintenance of a redox balance with minimal ROS production to sustain HIF-1 regulation and downregulation of antioxidants is one of the critical requirements of cancer cells. Accordingly, the metabolic switch to glycolysis has been ascribed as one of the adaptive mechanisms to reduce the level of ROS generated via OxPhos (36).

Next, in solid tumors, the presence of TME has been recognized as one of the major barriers that hinders successful tumor elimination by antitumor immune cells. TME is a complex medium, which influences and gets influenced by, the metabolic phenotype of cancer (43). The biochemical composition and the pH of the TME are primarily governed by the secretory/excretory products of cancer cells as well as the adjacent stromal cells or cancer-associated fibroblasts (CAFs). Emerging data indicate that CAFs play a pivotal role in the maintenance of tumor growth (44). CAFs have been known to utilize one of the metabolic products of cancer, the lactic acid or lactate. Removal of lactate by CAFs regulates/reduces chronic extracellular acidification. In addition, the utilization of lactate by CAFs *via* mitochondrial OxPhos to meet their energy demands reduces their demand for glucose, leading to increased glucose availability for cancer cells. Thus, CAFs indirectly facilitate glucose availability to fuel tumor metabolism. If tumor glycolysis is disrupted and lactate production is alleviated, the CAFs will rely on glucose metabolism leading to a competition with cancer cells for glucose uptake. Thus, disruption of tumor glycolysis will necessitate cancer cells to utilize mitochondrial-dependent OxPhos to meet their energy requirements. This in turn would deregulate the redox balance due to overly production of ROS. In addition, such an increase in intracellular ROS level along with a competition by CAFs for glucose consumption likely to enforce a metabolic pressure. Thus, dysregulation of tumor glycolysis could render cancer cells metabolically weak and sensitive to therapeutic interference.

**Figure 2:**
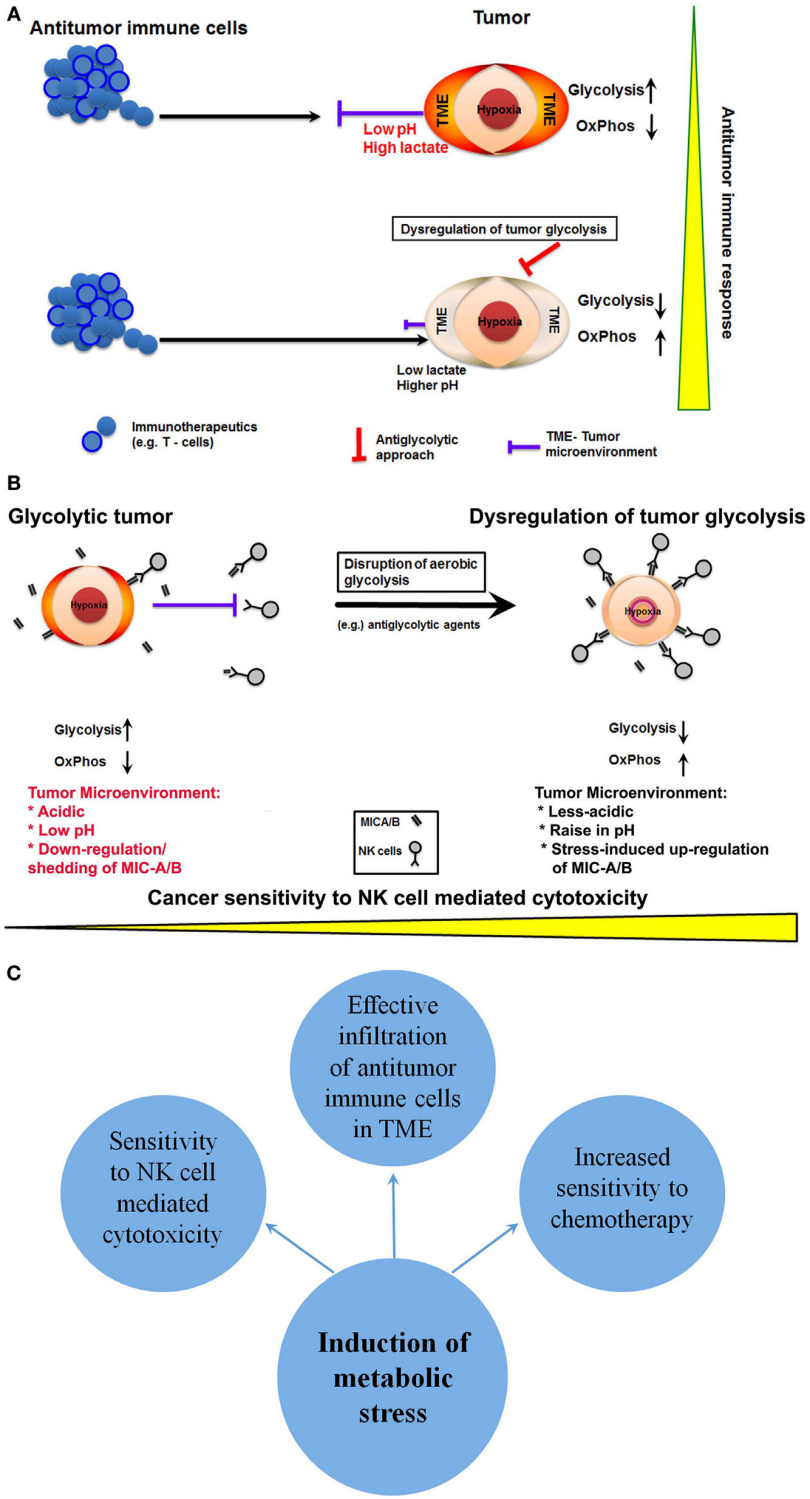
**Potential anticancer immunotherapeutic opportunities of dysregulation of tumor glycolysis**. **(A)** A schematic showing that dysregulation of tumor glycolysis alters tumor microenvironment that in turn could facilitate effective infiltration of antitumor immune cells. **(B)** Diagrammatic representation of dysregulation of tumor glycolysis to upregulate the stress-inducible surface ligands for further sensitization to natural killer (NK) cell-mediated cytotoxicity. **(C)** A schematic showing potential outcomes of induction of metabolic stress by dysregulation of tumor glycolysis.

From the immunotherapy perspective, the abrogation or reduction of lactate production that in turn reduces the acidification of TME is a desirable consequence for effective infiltration of therapeutics including immune cells. Noteworthy, low pH and increased acidification of TME are principal reasons for the lack of efficacy or loss of function of several therapeutics including chemotherapeutics and immunotherapeutics (45). Comprehensibly, reduced acidification would facilitate enhanced penetrability of antitumor immune cells such as T-cells or NK cells or novel therapeutics like CAR-T cells (Figure 2A). In fact, sporadic reports have indicated that disruption of tumor glycolysis to limit the accumulation of lactate in TME could promote antitumor immune response (6, 46). Thus, dysregulation of glycolysis in cancer cells could facilitate effective targeting of cancer by antitumor immune cells.

Next, the NK cells represent the first line of defense, and preclinical reports indicate that NK cell-mediated cytotoxicity affects cancer cells (13, 47). However, recognition of cancer cells by NK cells depends upon two critical factors; (a) the recognition of specific antigens known as NK group 2D ligands (NKG2DLs) on the cancer cell and (b) the infiltration through TME. As discussed above, the latter may be overcome by dysregulation of tumor glycolysis, which will alter the acidic TME into less-acidic medium enabling effective infiltration of NK cells. However, the NK cell recognition of cancer by specific NKG2D ligands such as ULBP, MIC-A/B relies on their level of expression on target cells. Paradoxically, cancer cells downregulate the expression of NKG2DLs. Besides the reduction in expression, cancer cells have also been known to cleave the extracellular domain of the ligands like MIC-A/B, and such cleavage results in the release of soluble ligands. These soluble cleaved-products bind with specific NK cell receptors resulting in the neutralization of NK cell activity (Figure 2B). Intriguingly, recent reports show that MIC-A/B is stress inducible and is upregulated during cellular stress, such as oxidative stress (48) or thermal stress (49). In fact, recent experimental evidence shows that metabolic perturbation induces the expression of MIC-A/B (50). Thus, interference with tumor glycolysis and subsequent metabolic stress is a potential inducer of MIC-A/B expression, which could render cancer cells sensitive to NK cell-mediated cytotoxicity (Figure 2B).

## Conclusion

Mounting evidence establish that tumor-specific alteration in energy metabolism could be the Achilles’ heel of cancer (51). It is also clear that glycolytic phenotype influences the TME. Particularly, the impediments like acidic and low pH that hinder efficacy of majority of therapeutics including infiltrating antitumor immune cells. Thus, dysregulation of tumor glycolysis has the potential to sensitize cancer cells to NK cell-mediated immunotherapy by the upregulation of stress-inducible NKG2DLs (MIC-A/B) and affect the acidity of TME rendering increased penetrability or infiltration of antitumor immune cells (Figure 2C). However, to achieve cancer-specific glycolytic dysregulation and to enhance the effectiveness of anticancer immunotherapeutics, it is imperative to overcome some major challenges. Selective inhibition of glycolysis in cancer cells but not of healthy cells is the primary requirement. In this context, recent preclinical evidences have indicated the feasibility of selective targeting of tumor glycolysis by small molecules that rely on cancer-specific upregulation transporters like MCT-1 (52, 53). Nonetheless, detailed clinical investigations are mandatory to ascertain the translational potential of such molecules and strategies. Next, emerging reports demonstrate that inhibition of glycolysis or glucose deprivation facilitates metabolic switch to OxPhos in some cancers and lead to aggressive phenotype (e.g., metastasis) (54). In such glycolytically impaired but OxPhos dependent cancer cells, therapeutic targeting of mitochondrial respiration using potential anti-mitochondrial or mitotropic agents could be a viable anticancer approach (35, 55). However, the impact of such metabolically altered phenotype in its sensitivity to anticancer immune therapeutics remains to be investigated. Nonetheless, the antiglycolytic approach-related changes in TME may still yield favorable outcomes with anticancer immune therapeutics.

Next, the dysregulation of tumor glycolysis needs to be achieved by a strategy or therapeutic that is less toxic to circumvent the problem of inadvertent or undesirable effects on NK cells’ efficacy. Similarly, one of the common causes of diminished antitumor immunity is the undesirable toxicity that emanates from prior treatments. This is prevalent in cases of prior chemotherapy as the effective dose of chemotherapy relies on maximum tolerated dose, whereas such doses are invariably toxic and affect the maturation or functional activation of immune cells (56). Thus, any agent employed to dysregulate tumor glycolysis should be sufficient to disrupt the metabolic process but not toxic. Indeed, it is preferred that such glycolytic inhibition does not kill cancer cells, as the goal is to sensitize cancer cells to immunotherapy, which in turn will enable us to expand the repertoire of antitumor immunity. Such low-dose chemotherapeutics have also been shown to enhance the effectiveness of anticancer immunotherapy (57). Thus, future studies on the selective dysregulation of tumor glycolysis to alter TME and the related therapeutic resistance could advance our ability to infiltrate and effectively target solid tumors by immunotherapeutics.

## Author Contributions

The author confirms being the sole contributor of this work and approved it for publication.

## Conflict of Interest Statement

The author declares that the research was conducted in the absence of any commercial or financial relationships that could be construed as a potential conflict of interest.
